# Challenges of coronavirus disease 2019 (COVID-19) in Lebanon in the midst of the economic collapse

**DOI:** 10.1017/ash.2021.244

**Published:** 2022-04-21

**Authors:** Nada K. Zahreddine, Sara F. Haddad, Anthony Kerbage, Souha S. Kanj

**Affiliations:** 1 Infection Control and Prevention Program, American University of Beirut Medical Center, Lebanon; 2 Division of Infectious Diseases, American University of Beirut Medical Center, Lebanon; 3 Department of Internal Medicine, American University of Beirut Medical Center, Lebanon

**Keywords:** COVID-19, Lebanon, Beirut blast, Pandemic, Economic collapse, Vaccine

## Abstract

Lebanon, one of the smallest countries in the Middle East, has held for decades a reputation for being the premier medical hub for patients from the Arab world as well as neighboring countries and for offering world-class treatment and advanced medical services. However, this once world-renowned healthcare sector is now facing a risk of imminent collapse as overlapping crises have struck the country since October 2019. In this article, we describe the concomitant political, economic, and financial challenges that Lebanon is facing, which have strained the national healthcare system and have undermined its ability to respond to the coronavirus disease 2019 (COVID-19) pandemic. We present an overview of the political instability, the Lebanese revolution with countrywide protests, as well as the devaluation of the Lebanese currency representing one of the worst economic meltdowns since the 19th century. We describe the unprecedented energy crisis the country is facing and the disastrous consequences of the Beirut port explosion of August 2020. We review the efforts and measures taken by different stakeholders to contain the COVID-19 pandemic amid the multifaceted challenges and the large exodus of healthcare personnel.

Lebanon, a country in the Middle East, is home to ∼6.8 million people, of whom ∼25% are Syrian, Palestinian, or of other origin. Despite its small area (10,453 km^2^ or 4,036 mi^2^), Lebanon continues to host the largest number of refugees relative to its national population.^1^ In the 1950s, Lebanon had a high national income founded by a strong banking system and a diversified successful economy based on tourism and commerce.^2^ These assets earned the country the nickname of “Switzerland of the Middle East.”^3^ Lebanon is an independent country, wedged between Syria and Israel, with many conflicts and crises, historically, that forced people to immigrate to and settle in Africa, the Americas, and Europe. The largest exodus happened during the 15-year civil war. However, after the war ended in 1990, many Lebanese returned, but some of them fled again during the 2006 war with Israel. As a result, the Lebanese diaspora today is nearly 3 times the size of Lebanon’s population.^4^ For the past 15 years, migration had been relatively stable until drastic multifaceted events took place and changed the outlook of the country. The devaluation of the currency due to the corrupt governance and the central bank’s policies, led to gradual economic collapse and triggered protests on the streets in October 2019. This activity culminated in what has been labeled the Lebanese Revolution and resulted in the resignation of the government.^5^ This was followed by the catastrophic Beirut port explosion in August 2020, which had a huge impact on many Lebanese citizens. These simultaneous crises have undermined Lebanon’s ability to respond to the coronavirus disease 2019 (COVID-1) pandemic. Here, we present an overview of the challenges faced by the Lebanese residents and their impact on the national COVID-19 response.

## Overview of the situation in Lebanon

### Economic collapse and political unrest

Early in 2018, the country started suffering a major economic and financial collapse due to the long-term political corruption and bad governance that triggered the Lebanese Revolution in October 2019.^6^ Hundreds of thousands of people went to the streets to protest, asking for the resignation of the government and political reforms. During the following 2 years, the national currency progressively lost 90% of its value, rendering more than half of the population below the poverty line with poor access to proper health care.^7^ Furthermore, banks faced liquidity problems, and limits were imposed on depositors for cash withdrawal. Concomitantly, private companies, including healthcare facilities, started to lay off employees because of financial difficulties. Subsequently, hundreds of employees and self-employed individuals voluntarily chose to leave the country to seek better jobs and life opportunities. The compounded Lebanese crises makes its situation unique compared to other global crises. Professionals who provide essential services (eg, doctors, nurses, engineers, academicians, and entrepreneurs) are the first to relocate in such situations.^4^ The trends in Lebanon are very worrisome. Recently, the World Bank warned against a brain drain in Lebanon; the economic crisis is thought to be 1 of the 3 most severe crises in the world since the 19th century. The mass migration is expected to cause permanent damage to the human capital that will make it very hard to recover. In 2021, the Lebanese Order of Physicians and the Order of Nurses estimated that ∼1,700 nurses and 1,000 of the 15,000 registered doctors had left the country since 2019.^8^ Others estimate that 20% of physicians would have left by the end of 2020. The current net migration rate for Lebanon in 2021 is −16.538 per 1,000 population, or 32.06% greater migration than in 2020. An estimated 300 persons are leaving the country every day.^9^

### August 2020 Beirut blast

**Fig. 1.**
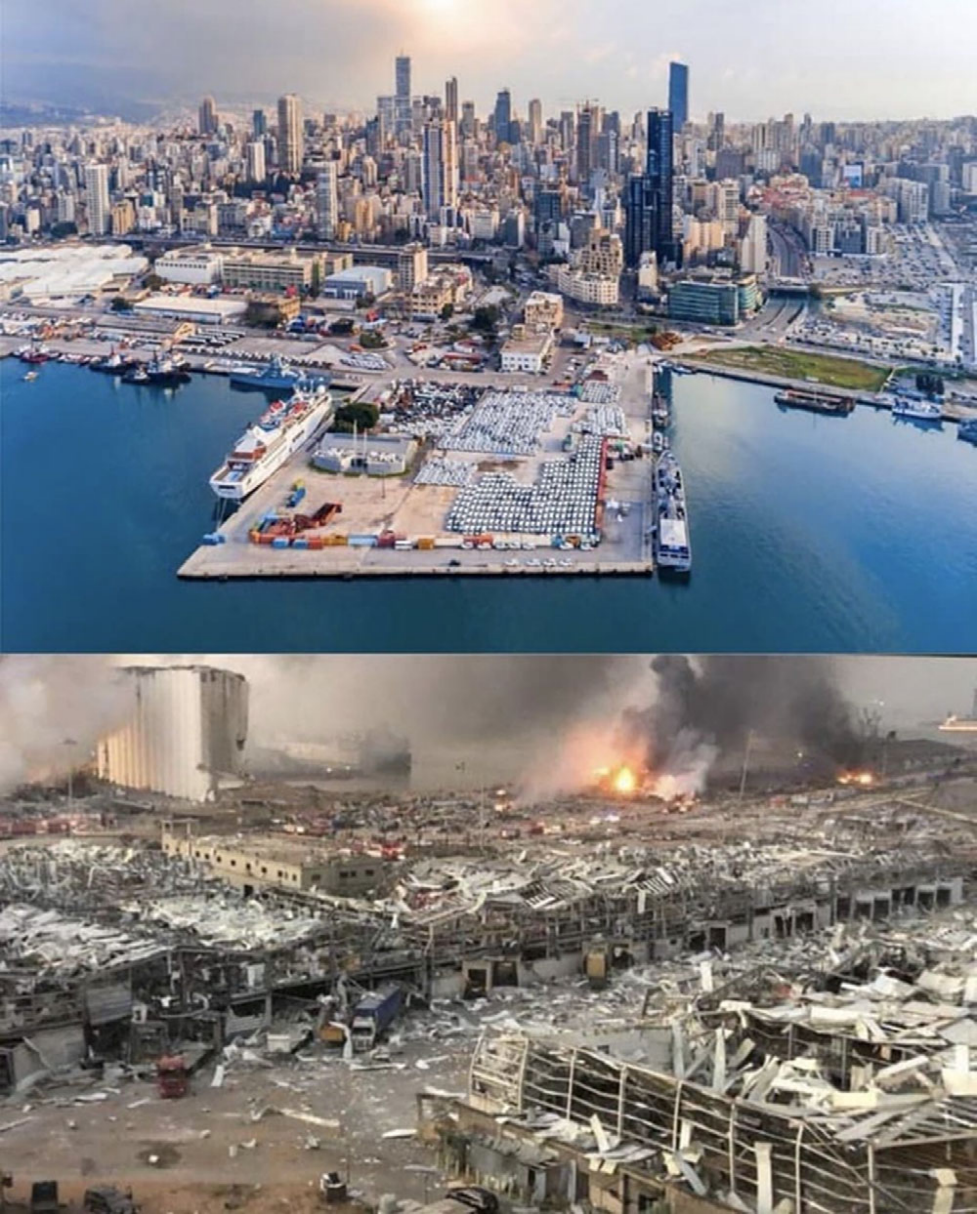
(a) A view of the Beirut port before the explosion of August 4, 2020. (b) A view of the massive damage of Beirut’s port caused by the explosion.^40^

**Fig. 2.**
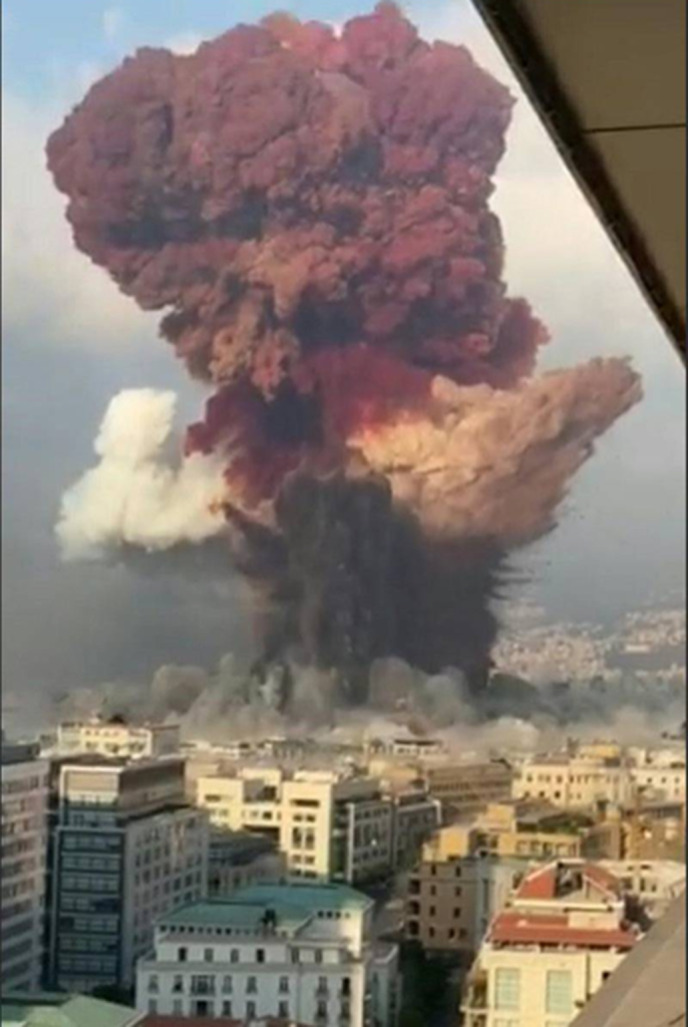
A screenshot from footage filmed from an office building the moment the explosion rocked Beirut on August 4.^41^

On August 4, 2020, a colossal explosion marked Beirut history and made the headlines all over the world (Fig. 1). The blast killed at least 220, injured 6,500, disabled 1,000, and displaced ∼300,000 persons.^10^ According to Lebanese authorities, it was ignited by the illegal storage of ∼2,750 tons of highly explosive ammonium nitrate stockpiled in the Beirut port. Experts estimated the size of the explosion to be the biggest nonnuclear blast of all time (Fig. 2)^11^. To date, governmental investigations have not revealed the political and/or military circumstances leading to this massive blast.

The deadly explosion devastated the healthcare system, which was already struggling to stay afloat. Hospitals were already experiencing tremendous challenges in terms of capacity and resources. They were prioritizing preparedness for the COVID-19 pandemic, and 209 cases were reported on the day of the explosion.^12^ According to the World Health Organization (WHO), on that day, 3 central hospitals sustained catastrophic damage and were forced to close immediately after the blast; 3 other hospitals had to reduce their capacity and 12 primary healthcare centers (PHCs) were severely damaged. At the port, 17 containers of personal protective equipment (PPE) and medical supplies were demolished, causing additional shortages in the medical sector.^13^ Healthcare personnel, including doctors and nurses, were also affected; many were severely injured and some died.

Shortly after the blast, several hospitals in Beirut reached full capacity and emergency entrances were inundated.^14^ Injured people were treated in the hallways and on the streets without any attention to infection control measures. Healthcare workers (HCWs) were racing to save lives, with little attention to wearing masks or washing hands. Their priority was to provide immediate medical and surgical care to the victims. COVID-19–related infection control practices were difficult to maintain as people rushed to help the injured, overlooking physical distancing measures amid a very difficult humanitarian situation. When dealing with mass casualty management during a pandemic, it is important to consider that each trauma victim could potentially be infected. However, this factor was totally overlooked due to the size and impact of the explosion. Many emergency departments ran out of PPE and other medical supplies.^15,16^

The Lebanese government was unable to cope with the damage to the healthcare sector. However, the regional and international aid responses were immediate. Field hospitals and mobile clinics were dispatched on site, and medical supplies were sent to Lebanon to help deal with the blast consequences. Strong collective support from Lebanese citizens, local organizations, and international nongovernmental organizations (NGO) delivered essential support to the victims and helped with reconstruction activities.^17^

### Energy crisis and fuel shortages: The last straw

Lebanon’s struggle with electricity goes back to the 1975–1990 civil war, due to the authorities’ failure to rehabilitate its main power plants, which are only capable of producing electricity to cover an average of 2 hours per day. As a result, the population relies on their own generators or private neighborhood suppliers to cover the remaining 22 hours. However, in the wake of the ongoing economic crisis and lack of foreign currency, the Central Bank of Lebanon imposed restrictions on imports of subsidized fuel, which created extreme shortages in fuel, gas, diesel, and electricity.

The energy crisis, which led to extended power cuts across the country, reached its peak in August 2021. As a result, medical centers in Lebanon sent an alarming message to the international community about an imminent disaster resulting from a forced shutdown due to fuel and electricity shortages. The shutdown meant that ventilators and other lifesaving medical devices might cease to operate and patients living on respirators might face immediate death. Fortunately, fuel suppliers, the United Nations, the WHO, the World Bank, and others responded in due time and ensured fuel supplies sufficient for a short period to cover hospital demands. Nevertheless, major reforms against corruption are necessary for Lebanon to obtain grants and international loans to recover from the worst financial crisis in its history. Recently, the country experienced a complete electricity blackout for several days, after which the power grid was restored to service as it was before the outage.

## The COVID-19 pandemic: The Lebanese experience

### A timeline of the outbreak

In February 2020, the first COVID-19 case was confirmed in a patient arriving from Iran.^18^ In the following months, the public health efforts led by the Ministry of Public Health (MOPH) succeeded in containing severe acute respiratory coronavirus virus 2 (SARS-CoV-2) transmissions in the country. Between 1 and 182 cases were identified daily from March until July 2020.^19^ Schools, shopping malls, and restaurants were closed as early as February 29, 2020, but pharmacies and supermarkets remained open. Travelers arriving in Lebanon through the airport, by sea, and via land borders were required to provide a negative PCR test prior to entering the country and to take a second PCR test upon arrival.^20^ Due to the increasing number of cases, on March 15, the Lebanese authorities imposed a nationwide rigid lockdown enforced by the military.

**Fig. 3.**
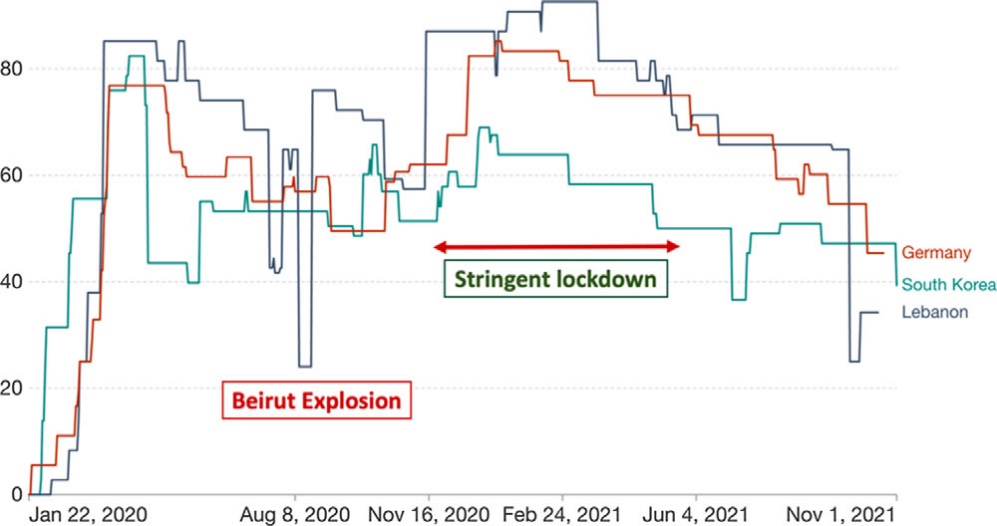
Oxford COVID-19 stringency index in Lebanon compared to South Korea and Germany. The COVID-19: stringency index is a composite measure based on 9 response indicators including school closures, workplace closures, and travel bans, rescaled to a value from 0 to 100 (100 = strictest). If policies vary at the subnational level, the index is shown as the response level of the strictest subregion.

Figure 3 shows the different lockdown measures imposed by the government during the pandemic (Fig. 3). The OXFORD COVID-19 Government Response Stringency index is calculated based on specific measures such as school and workplace closures, restrictions on public gatherings, and transportation.^21^ With an exception around the dates of the Beirut blast, the lockdown measures enforced by the Lebanese government were somehow effective compared to other countries known to have handled the pandemic successfully, such as Germany and South Korea.

In May 2020, the overall testing rate was 10,000 tests per 1 million population, which was comparable to global rates.^22^ Approximately 129 laboratories were certified to conduct COVID-19 tests using the real-time reverse transcription polymerase chain reaction (PCR). Prompt isolation of clusters coupled with massive testing were initially effective in containing transmission. Available hospital beds (acute and chronic) in Lebanon were reported at 2.73 per 1,000 people in 2017.^23^ Some of these were designated as COVID-19 beds, particularly in large university hospitals.

However, despite the early success, the country witnessed a rise in COVID-19 cases in July 2020 and then a sharp increase in cases following the Beirut blast, surpassing 680 daily cases by the end of August 2020.^19^

More than 1,000 cases were confirmed each day in September,^19^ outnumbering the capacity of the beds allocated for the care of COVID-19 patients in most hospitals. This presented a serious challenge to the national COVID-19 committee appointed by the MOPH. Infections grew out of control as the end of the year approached, and infections reached their peak in January 2021, surpassing 6,000 daily cases. The healthcare system was on the verge of collapse as the cumulative number of cases reached 119,549, marking the beginning of the 3 deadliest months in the country.^24^ Mortality attributed to COVID-19 complications increased, with 62% of all reported deaths being among patients aged >70 years.^20^

**Fig. 4.**
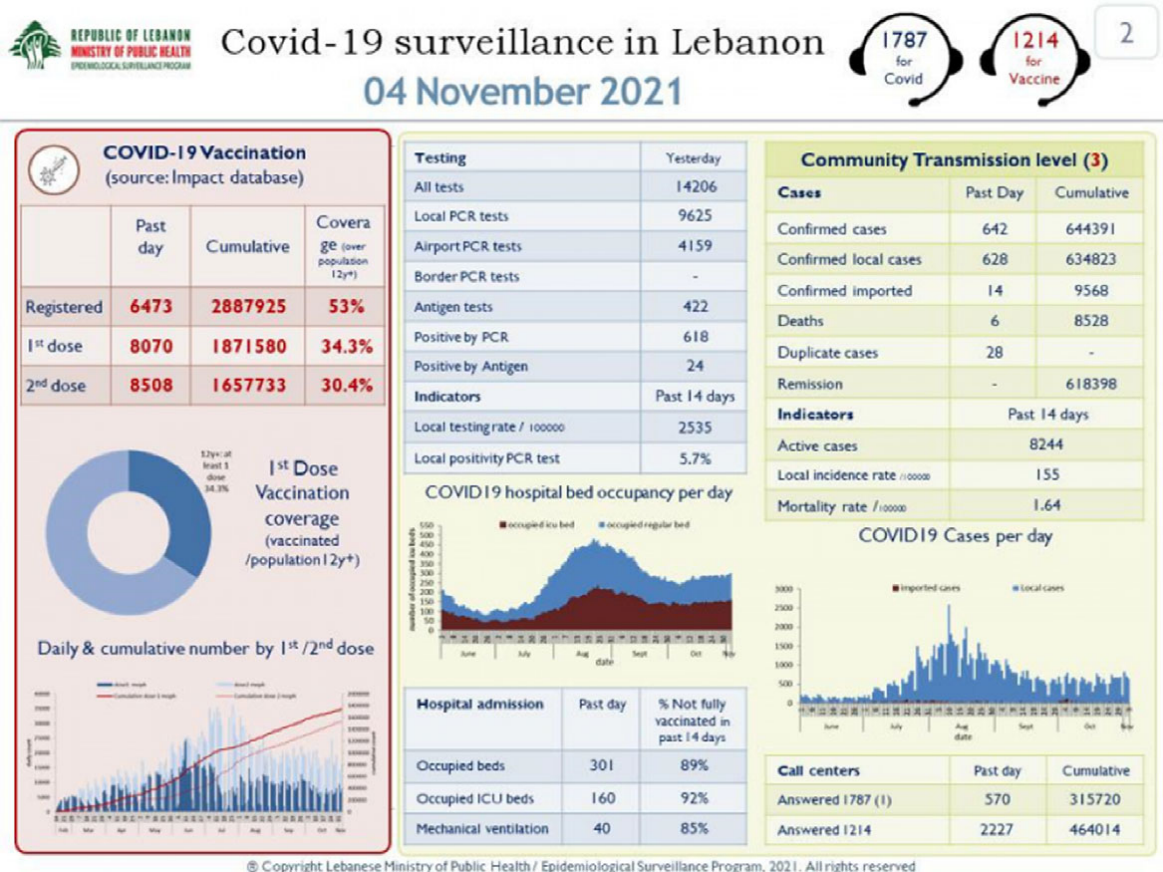
Example of daily COVID-19 statistics posted online by the Lebanon Ministry of Public Health.

Daily reports were posted online at the MOPH website (Fig. 4). These data included daily new confirmed cases, among local citizens and travelers, as well as the percentage of positive tests among all tested, the number of deceased patients, and other relevant data. However, it was believed that significant underreporting occurred; many patients with classic symptoms were not tested because of the cost of PCR tests. It was assumed that 1 positive case in a household meant that most household members would be infected. The average family in Lebanon has 4–6 members. Therefore, many experts estimated that the cumulative number of 620,000 in early October 2021 might reflect between 2 and 3 million cases, or ∼50% of the Lebanese population.

During these months, all elective admissions stopped, but hospitals continued to receive oncology patients and urgent cases. Medical equipment, tools, and devices needed to manage patients became scarce and significantly expensive, which constituted a major constraining factor to the healthcare sector.^24^

Both public and private hospitals suffered in the same way during the pandemic. When hospitals reached full bed capacity, emergency departments (EDs) became inpatient units, managing patients in hallways and entrances. Accordingly, boarding patients in the ED resulted in significant congestion, which is often associated with poor outcomes.^25^

Outbreaks of multidrug-resistant organisms were feared, including the emergence of a *Candida auris* outbreak at the American University of Beirut Medical Center (AUBMC), an infection which had never been reported from Lebanon before the COVID-19 pandemic.^26^

### Response efforts

Despite the many political, financial and economic challenges, the MOPH managed the outbreak effectively. In addition to leading the national COVID-19 response and deciding on restrictions and lockdown measures, their work included planning awareness campaigns, investigations and contact tracing.^27^ At some points during the pandemic, they provided free tests and financial support to remote areas away from big cities and underresourced villages. The Lebanese Red Cross, NGOs and major hospitals were mobilized and launched massive testing campaigns across the country. Municipalities were also trained to locate clusters; they helped enforce lockdown measures and they supervised burial rituals in the country.^28^ Conversely, efforts to manage the impact of the COVID-19 pandemic on mental health remained inadequate despite a few efforts by the health authorities.^29^

The support of WHO and UNICEF to the healthcare system during the pandemic was exceptionally valuable. These organizations provided training to staff in public hospitals, delivered PPE and medical supplies, and closely followed the management of the crisis.^30,31^ One tertiary-care hospital, Rafik Hariri Governmental University Hospital (RHUH), admitted all COVID-19 cases at the beginning of the pandemic and was entirely supported by the WHO.

In addition to governmental hospitals, many private hospitals shouldered the burden of managing the pandemic and helped in awareness-raising campaigns. AUBMC was one of the private hospitals that played a major role in managing the pandemic and served as a model for other hospitals in the country and the region. Through a project funded by USAID, the AUBMC led a national project for COVID-19–related infection control training and education of HCWs in 10 peripheral hospitals in Lebanon. PPE and medical supplies were also provided by USAID to these hospitals. Similarly, and in response to global shortages, AUBMC obtained locally manufactured alcohol-based hand solutions that fulfilled the WHO requirements.

**Fig. 5.**
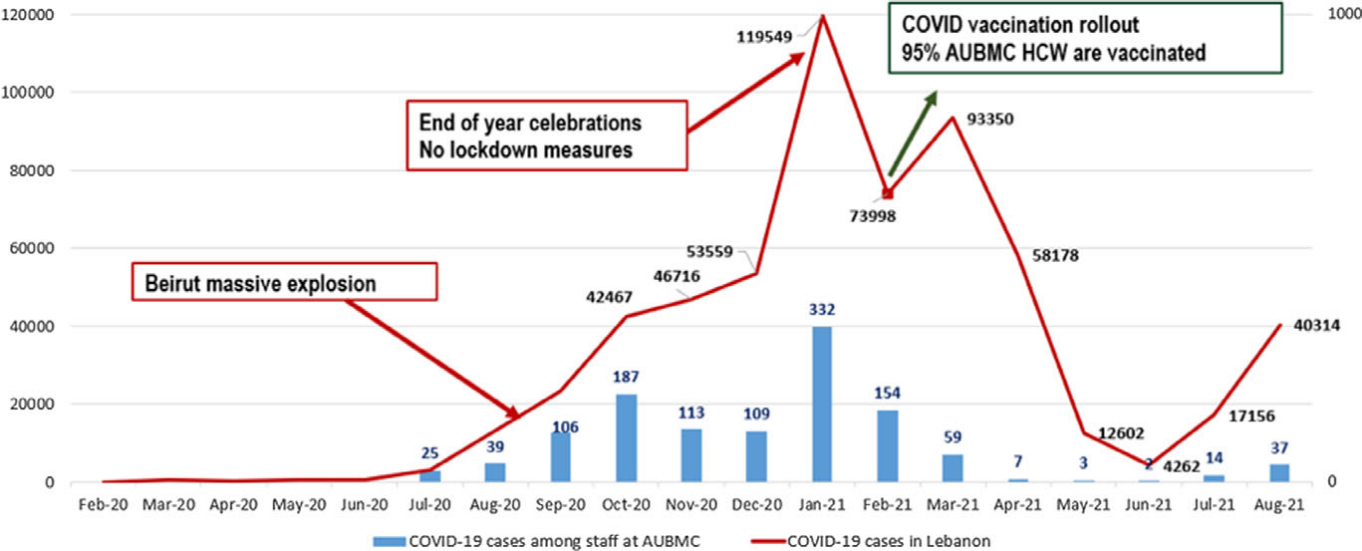
Incidence of COVID-19 among AUBMC HCWs compared to incidence at the national level in Lebanon (August 2021). Note. AUBMC, American University of Beirut Medical Center; HCW, healthcare worker.

Nevertheless, during the peak of the pandemic, the risk of SARS-CoV-2 transmission increased among hospital employees. For example, at AUBMC, 26% of the 3,921 employees had been infected by February 2021. The numbers followed the national trends and were marked by a significant increase following the Beirut explosion and another increase following the end of year celebration where lockdown measures were lifted (Fig. 5).

Despite all the challenges in Lebanon, research activities were conducted over the past 2 years, and dozens of COVID-19–related scientific papers have been published. Topics have been related to patient management, infection outbreaks, infection control, social and psychological impacts, as well as vaccination rollouts.

### COVID-19 vaccination in Lebanon

**Fig. 6.**
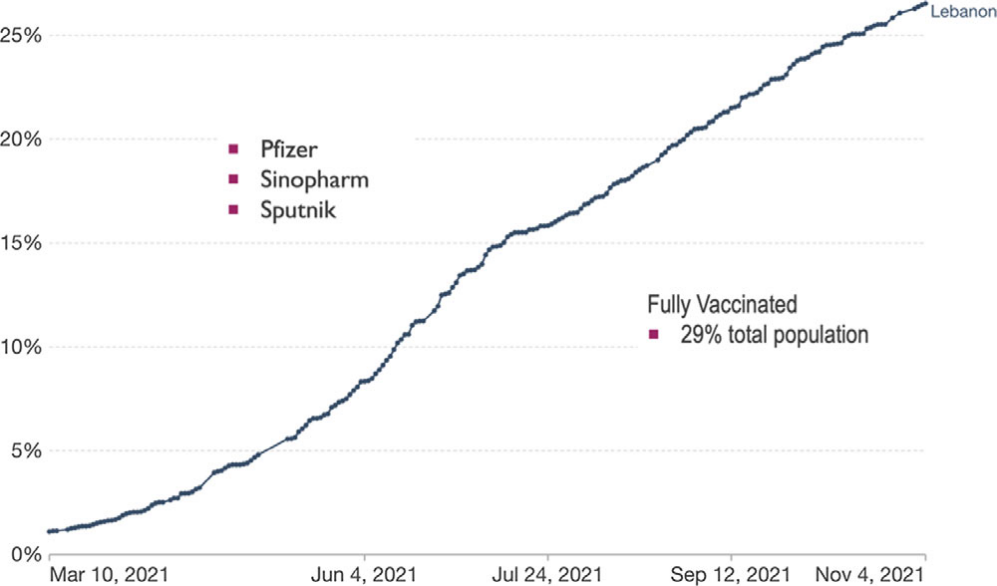
Vaccination rollout in Lebanon (first dose of the vaccine). Share of people who received at least one dose of COVID-19 vaccine: Total number of people who received at least one vaccine dose, divided by the total population of the country.

Amid all the challenges, in February 2021, the MOPH announced the purchase of the Pfizer-BioNTech COVID-19 vaccines, after obtaining an exceptional approval of US$34 million from the World Bank to vaccinate free of charge ∼2 million Lebanese residents.^32^ Initially, people aged >75 years, HCWs, and patients with chronic diseases were vaccinated. The vaccination campaign was launched in major university hospitals in Beirut. They were overseen by the MOPH, and the COVAX platform was adopted for registrations. Further quantities of COVID-19 vaccines started to arrive, including AstraZeneca vaccines (Fig. 6).

Subsequently, the private sector intervened to speed-up the vaccination rollout. Their aim was to reopen businesses that were struggling through the unprecedented financial collapse. Sputnik V and Sinopharm were also approved for use in Lebanon and were sold and administered by the private sector.

The vaccination rollout in Lebanon is considered successful. To date, 3,346,000 doses of COVID-19 vaccines have been administered to 1.58 million people, estimated to represent ∼29% of the residents in Lebanon. Adverse reactions following vaccinations were reported through the MOPH platform, and 94.1% were considered nonserious.^33^

In October 2021, the daily reported COVID-19 cases considerably decreased with a noticeable reduction in mortality rates mostly thought to result from the successful vaccination rollout in Lebanon.^20^ In parallel, the daily death toll decreased to 7–8 deaths in September 2021, compared to 80 deaths in February 2021, and hospital beds became again available for patients with COVID-19.^19^

### Circulation of COVID-19 variants

COVID-19 variants started to make the headlines as early as December 2020. New genetic lineages of SARS-CoV-2 emerged and circulated around the world. The B.1.617.2 δ (delta) variant was reported as a variant of concern by the WHO.^34^ According to the CDC, the δ variant causes more infections and spreads faster than earlier forms of the virus.^35^ Approved vaccines such as Pfizer-BioNTech or AstraZeneca, are thought to be effective against these variants.^36^ In Lebanon, the α (alpha) variant swept rapidly across the country and caused the largest wave, which peaked in January 2021.^37^ This variant was detected in Gambia and was transmitted by travelers returning to Lebanon in December 2020. Currently, the δ variant has replaced the α variant. In a recent study, 11 countries (Germany, Switzerland, Turkey, etc) had perfectly matching genomes with 40 Lebanese genomes, likely due to the common travel from and to these countries.^38^

## Mounting challenges

Unfortunately, the situation in the country is not promising and the Lebanese people are not seeing a light at the end of the tunnel. The workforce is exhausted; experienced HCWs, other professionals, and students continue to leave by the thousands every month seeking better lives and job opportunities. The incessant humanitarian disasters continue to heavily weigh on the country. More than half of the Lebanese now live below the poverty line. The international community is aware of the potential repercussions this situation could have on neighboring countries and Europe. It remains to be seen whether the new government, appointed on September 10, 2021, can implement the needed reforms and restore the country’s reputation and status. As we conclude this paper, armed clashes erupted in Beirut, close to the former green line of the 1975–1990 civil war, aggravating the instability and anticipating more violence in the future.^39^

In conclusion, the ongoing COVID-19 pandemic has been devastating to most countries. In Lebanon, the challenges have been exacerbated by the economic crisis, political turmoil, and the Beirut blast. The exodus of healthcare providers, among others, intensified the challenges to those remaining, who have had to deal with increased workloads at a greatly reduced income. The COVID-19 vaccination rollout continues with the support of international agencies. The national implementation of infection control measures, ongoing training, and education of HCWs proved instrumental in efforts to contain the pandemic.

Despite an unprecedented financial collapse, Lebanon mobilized the public and private health sectors as well as the military to help manage the pandemic. In addition, HCWs learned valuable lessons on the management of mass casualties in the setting of a pandemic. However, in view of the persistence of the global pandemic, the Lebanese authorities must remain vigilant and monitor the international reports for the emergence of new COVID-19 variants of concern.

## References

[1] Global trends: forced displacement in 2017. United Nations High Commissioner for Refugees website. https://www.unhcr.org/5b27be547.pdf. Published 2017. Accessed October 16, 2021.

[2] Lains P. Joerg Baten , ed., A History of the Global Economy: 1500 to the Present. (Cambridge UK: Cambridge University Press; 2016:xiv–369.99/$119.99). Econ Hist Rev 2017;70:366–368.

[3] Young M. The Switzerland of the Middle East? *Executive Magazine* website. https://www.executive-magazine.com/business/the-switzerland-of-the-middle-east. Published 2007. Accessed October 16, 2021.

[4] Vohra A. Lebanon is in terminal brain drain. *Foreign Policy* website. https://foreignpolicy.com/2021/08/09/lebanon-terminal-brain-drain-migration/. Published 2021. Accessed October 18, 2021.

[5] Lebanon: why the country is in crisis. BBC News website. https://www.bbc.com/news/world-middle-east-53390108. Published 2020. Accessed October 16, 2021.

[6] The making of Lebanon’s October Revolution. *The New Yorker* website. https://www.newyorker.com/news/dispatch/the-making-of-lebanons-october-revolution. Accessed October 16, 2021.

[7] Welle (www.dw.com) D. Lebanon: dollars shield some from hyperinflation crisis. DW Akademie website. https://www.dw.com/en/lebanon-dollars-shield-some-from-hyperinflation-crisis/a-58843383. Published 2021. Accessed October 16, 2021.

[8] Lebanon hospitals under threat as doctors and nurses emigrate. *The National* website. https://www.thenationalnews.com/world/mena/lebanon-hospitals-under-threat-as-doctors-and-nurses-emigrate-1.1077418. Accessed October 16, 2021.

[9] Lebanon net migration rate, 1950–2021. Macrotrends website. https://www.macrotrends.net/countries/LBN/lebanon/net-migration. Published 2021. Accessed October 16, 2021.

[10] Al-Hajj S , Dhaini HR , Mondello S , Kaafarani H , Kobeissy F , DePalma RG. Beirut ammonium nitrate blast: analysis, review, and recommendations. Front Public Health 2021;9:657996. 34150702 10.3389/fpubh.2021.657996 PMC8212863

[11] Beirut explosion: Lebanon’s government “to resign” as death toll rises. BBC News website. https://www.bbc.com/news/world-middle-east-53720383. Published 2020. Accessed October 16, 2021.

[12] Sayed MJE. Beirut ammonium nitrate explosion: a man-made disaster in times of the COVID-19 pandemic. *Disaster Med Public Health Prep* 2020. doi: 10.1017/dmp.2020.451. PMC7985624 33203497

[13] Beirut explosion situation report no. 2 UNFPA Lebanon website. https://lebanon.unfpa.org/en/publications/beirut-explosion-situation-report-no2. Published 2020. Accessed February 14, 2021.

[14] Mansour HA , Bitar E , Fares Y , et al. The Beirut Port explosion: injury trends from a mass survey of emergency admissions. Lancet 2021;398:21–22. 10.1016/S0140-6736(21)01246-0 34217389

[15] Hallal A , Andraos R , Saad GA , Boyajian T , Hoballah J. Mass casualty management during a pandemic surge: the American University of Beirut Medical Center experience. Semin Vasc Surg 2021;34:51–59. 34144748 10.1053/j.semvascsurg.2021.04.005

[16] El Sayed MJ , Hitti EA , Cheaito MA , Davis T , Kazzi AA. mass casualty management in the emergency department: lessons learned in Beirut, Lebanon—part II. *Mediterr J Emerg Med Acute Care* 2020. doi: 10.52544/2642-7184(1)3003. 37838490

[17] Lebanon: 13,000 Beirut blast victims helped through EU–NGO partnership. ReliefWeb website. https://reliefweb.int/report/lebanon/lebanon-13000-beirut-blast-victims-helped-through-eu-ngo-partnership. Published 2021. Accessed October 16, 2021.

[18] Lebanon Ministry of Public Health website. http://www.moph.gov.lb. Accessed October 16, 2021.

[19] Lebanon COVID: 632,781 cases and 8,413 deaths. Worldometer website. https://www.worldometers.info/coronavirus/country/lebanon/. Published 2021. Accessed October 16, 2021.

[20] Monitoring of COVID-19 infection in Lebanon, October 26, 2021. Lebanon Ministry of Public Health website. http://www.moph.gov.lb/en/Media/view/43750/monitoring-of-covid-19. Published October 26, 2021. Accessed October 27, 2021.

[21] Lebanon: coronavirus pandemic country profile. Our World in Data website. https://ourworldindata.org/coronavirus/country/lebanon#global-cases-in-comparison-how-are-cases-changing-across-the-world. Accessed October 29, 2021.

[22] Testing for COVID-19 in Lebanon. *Executive Magazine* website. https://www.executive-magazine.com/economics-policy/healthcare/coronavirus/testing-for-covid-19-in-lebanon. Accessed October 18, 2021.

[23] Lebanon Hospital Beds. Trading Economics website. https://tradingeconomics.com/lebanon/hospital-beds-per-1-000-people-wb-data.html. Accessed October 16, 2021.

[24] Bizri AR , Khachfe HH , Fares MY , Musharrafieh U. COVID-19 pandemic: an insult over injury for Lebanon. J Community Health 2021;46:487–493. 32661861 10.1007/s10900-020-00884-y PMC7358300

[25] Derlet RW , Richards JR. Ten solutions for emergency department crowding. West J Emerg Med 2008;9:24–27. 19561699 PMC2672221

[26] Allaw F , Kara Zahreddine N , Ibrahim A , et al. First *Candida auris* outbreak during a COVID-19 pandemic in a tertiary-care center in Lebanon. Pathog Basel Switz 2021;10:157. 10.3390/pathogens10020157 PMC7913166 33546137

[27] Latest COVID statistics. Lebanon Ministry of Health website. https://corona.ministryinfo.gov.lb/. Accessed October 16, 2021.

[28] Kerbage A , Matta M , Haddad S , et al. Challenges facing COVID-19 in rural areas: an experience from Lebanon. Int J Disaster Risk Reduct 2021;53:102013. 33318917 10.1016/j.ijdrr.2020.102013 PMC7726740

[29] Mental Health as a Priority for UN in Lebanon during COVID-19. United Nations in Lebanon website. https://lebanon.un.org/en/103974-mental-health-priority-un-lebanon-during-covid-19. Accessed October 22, 2021.

[30] UNICEF is providing supplies and technical support to fight COVID-19 in Lebanon. UNICEF website. https://www.unicef.org/mena/press-releases/unicef-providing-supplies-and-technical-support-fight-covid-19-lebanon. Accessed October 16, 2021.

[31] Lebanon explosion: update for partners, August 18, 2020. WHO website. https://www.who.int/emergencies/who-lebanon-partners-update-18august2020.pdf?ua=1. Published August 18, 2020. Accessed October 16, 2021.

[32] World Bank supports first COVID-19 vaccine rollout in Lebanon. World Bank website. https://www.worldbank.org/en/news/press-release/2021/01/21/world-bank-supports-first-covid-19-vaccine-rollout-in-lebanon. Accessed October 16, 2021.

[33] Rita K , Abeer Z , Katia I , Myriam W. Adverse Events Following Immunization Monitoring-Covid-19 Vaccines-Lebanon. Lebanon Ministry of Public Health website. http://www.moph.gov.lb. Accessed October 16, 2021.

[34] Tracking SARS-CoV-2 variants. WHO website. https://www.who.int/emergencies/emergency-health-kits/trauma-emergency-surgery-kit-who-tesk-2019/tracking-SARS-CoV-2-variants. Accessed October 16, 2021.

[35] Coronavirus disease 2019 (COVID-19). Centers for Disease Control and Prevention website. https://www.cdc.gov/coronavirus/2019-ncov/variants/delta-variant.html. Published 2020. Accessed October 16, 2021.

[36] Effectiveness of COVID-19 vaccines against the B.1.617.2 (delta) variant. *N Engl J Med* 2021;385:585–594. 10.1056/NEJMoa2108891 PMC8314739 34289274

[37] Merhi G , Trotter AJ , Martins L de O , et al. Replacement of the alpha variant of SARS-CoV-2 by the delta variant in Lebanon between April and June 2021. *MedRxiv* 2021. doi: 10.1101/2021.08.10.21261847v1. PMC9455693 35876490

[38] Feghali R , Merhi G , Kwasiborski A , Hourdel V , Ghosn N , Tokajian S. Genomic characterization and phylogenetic analysis of the first SARS-CoV-2 variants introduced in Lebanon. PeerJ 2021;9:e11015. 34611501 10.7717/peerj.11015 PMC8447710

[39] Hubbard B , Santora M. Deadly clashes in beirut escalate fears over Lebanon’s dysfunction. *The New York Times* website. https://www.nytimes.com/2021/10/14/world/middleeast/beirut-lebanon.html. Published October 14, 2021. Accessed October 22, 2021.

[40] Mabhiza L. Lebanon blast death toll reaches 157. *Mbare Times* website. https://mbaretimes.com/2020/08/lebano-157/. Published August 6, 2020. Accessed November 3, 2021.

[41] Beirut blast: what do we do with the world’s ammonium nitrate? *The National* website. https://www.thenationalnews.com/opinion/comment/beirut-blast-what-do-we-do-with-the-world-s-ammonium-nitrate-1.1062306. Accessed October 29, 2021.

